# Factors affecting men’s support for the use of the contraceptive implant by their female intimate partners

**DOI:** 10.1186/s40834-020-00140-7

**Published:** 2020-11-23

**Authors:** Kim Jonas, Moira Kalichman, Seth Kalichman, Chelsea Morroni, Catherine Mathews

**Affiliations:** 1 Health Systems Research Unit, South African Medical Research Council, Cape Town, South Africa; 2 Adolescent Health Research Unit, Department of Psychiatry and Mental Health, University of Cape Town, Cape Town, South Africa; 3 Department of Psychology, University of Connecticut, Storrs, Mansfield, CT USA; 4 Liverpool School of Tropical Medicine, Liverpool, UK; 5 Women’s Health Research Unit, School of Public Health and Family Medicine, University of Cape Town, Cape Town, South Africa; 6 Botswana-University of Pennsylvania Partnership, Gaborone, Botswana

**Keywords:** Long-acting reversible contraceptives (LARCs), Contraceptive implant, Implant, Implanon, Implanon NXT, Family planning, Men’s support, Men

## Abstract

**Background:**

Family planning services have been available at no cost in the public health settings of South Africa since 1994, and now include the long-acting reversible contraceptives (LARCs) namely, the contraceptive implant and intra-uterine device (IUD). However, the uptake of LARCs has been declining in the recent years and little is known about the cause of the decline. In many relationships, men may influence their female intimate partner’s contraceptive choices. Thus, men’s involvement in reproductive health decisions and family programming may improve their support for contraceptive use, including the LARC use by their female intimate partners. This study investigated factors affecting men’s support for the use of contraceptive implant by their female intimate partners.

**Methods:**

A quantitative, cross-sectional survey was conducted among adult men attending a public, primary health clinic in Cape Town, South Africa. Using a structured questionnaire, we measured men’s knowledge, awareness and support of, and attitudes towards use of the contraceptive implant by their female intimate partners. Data were analyzed using SPSS version 25.

**Results:**

The sample included 65 men with a mean age of 31.2 years. Most (76.6%) believed that both men and women should be responsible for family planning. Support for general contraception use by their female intimate partners was prevalent at 80.0%, but only 33.9% reported that they would like their partners to use the implant in the future, while 35.6% were unsure and 30.5% did not support their partner’s use of the implant. Factors significantly associated with men’s support of their partner’s future use of the contraceptive implant included men’s reports that their partner wished to have another child in future, knowledge that the implant is safe for use by women who have not had children, knowledge that the implant can effectively prevent pregnancy for 3 years, and a positive attitude towards the implant’s long-lasting effectiveness.

**Conclusion:**

Improving men’s knowledge of, and attitudes toward the contraceptive implant might increase their support for their partner’s use of the implant, which in turn might promote uptake of the implant among women. The findings of our study suggest the importance of actively engaging men in reproductive health and family planning programs.

**Supplementary Information:**

The online version contains supplementary material available at 10.1186/s40834-020-00140-7.

## Introduction

Family planning services have been available at no cost in public health services in South Africa since 1994, and now include the long-acting reversible contraceptives (LARCs) namely, the contraceptive implant and intra-uterine device (IUD). The Department of Health’s (DoH) policy on contraception and fertility planning, updated in 2014, expanding the method mix to give women greater choice for family planning [1]. According to the policy, “the methods of contraception were expanded to cover an increased access to LARC methods with with specific consideration of: increasing access to the copper intrauterine device (Cu IUD), with antibiotics at the time of insertion, and introduction of a single-rod progestogen implant…” [1]. The contraceptive implant, Implanon NXT, was the most common LARC on offer in South African public health services at no cost. It is a subdermal single-rod implant containing 68 mg of the progestin etonogestrel, which is highly effective in preventing pregnancy for a period of 3 years [2–4]. Despite the robust and supportive policies related to LARCs in the public sector, the use of the contraceptive implant among women is limited [5–7].

Among women, limited knowledge and awareness of the different contraceptive methods, including the implant, have been identified [7]. Additionally, concerns, myths and misconceptions about side effects and risks associated with the implant contribute to the limited uptake among women [8–10]. Research conducted among men on family planning use and support remains insufficient globally [11]. The few studies that have been conducted among men, show that male partner support for family planning influences women’s behaviour towards the use of modern contraceptives [11–14]. One study has been undertaken among women showing that beliefs about and perceptions of the implant’s side effects negatively influence men’s support for its use by their female intimate partners [15]. Another South African study found that women report lack of male partner support for the use of the implant as one of the reasons for early removal and discontinuation of use [16].

This study aims to describe the specific factors affecting men’s support for the future use of the contraceptive implant by their female intimate partners. Such an understanding can complement the women’s perceptions of male partner support for family planning use and help design specific interventions to ameliorate the challenges affecting the implant use among women. Improving the implant uptake may significantly reduce unintended pregnancies which are highly prevalent among adolescents and adults in the country [7, 17]. Ultimately, this study hopes to contribute towards the intervention program development for the reduction of unplanned and or unintended pregnancies, as well as the unmet need for contraception in the country.

## Methods

### Research design and study setting

This quantitative, cross-sectional survey was conducted among adult men attending a public, primary health clinic in Cape Town, South Africa. The clinic was selected because it is a typical public clinic in a poor, peri-urban setting. It offered a range of public primary health services with a primary focus on STI, HIV and Tuberculosis (TB) services, including other sexual and reproductive health services. It is worth noting that historically, men poorly attend health care facilities and often delay seeking health care services when they are ill for various reasons, such as discomfort with female staff nurses, clinics being full of women, and fear of being labelled as “weak” according to gender norms [18–20]. Consequently in South Africa, “men only” clinics were initiated to help attract more men seeking health care service, particularly for STI testing and treatment including HIV services. This specific clinic, though not a “specialized” male clinic, it also had a “men only” section for STI treatment with dedicated male providers. Despite this effort, there were still few men attending the clinic.

### Participant sampling

All men aged 18 years and above who were attending the clinic between February 2015 to February 2016 for sexual and reproductive health related services, including HIV and TB testing and treatment were invited to participate in this study. Although this study was conducted in a public health clinic which also included a “men only” section, very few men visited the clinic. Additionally, to be invited in the study, men had to be able to read and write in English or isiXhosa; not psychologically and or visually impaired. Hence, only 71 men were approached and recruited for this study over this period. Of the 71 sampled participants, 65 agreed to participate and completed the self-administered questionnaire. Those who declined to participate cited time constraints as the main reason.

### Data collection tool

A paper-pencil questionnaire format, requiring approximately 10 min for completion, was used to collect data. Participants could complete it in their preferred language (either English or Xhosa). Because of the sensitive nature of the questions, we offered self-administered survey completion to give participants privacy to answer the questions as truthfully as possible.

Demographic questions included marital or relationship status, and whether they had main or casual sex partners, or both. A main sexual partner was defined as a sexual partner an individual has for six or more months and included wives while a casual partner was defined as a “secret” sexual partner an individual has sex with occasionally and included the “one-night-stands”.

We included questions on partner’s use of family planning methods, as well as knowledge and awareness of, and outcome expectancies related to the contraceptive implant. We also included questions about the number of pregnancies for the female intimate partner, and fertility intentions. We asked whether participants had heard of each of the different types of contraceptive methods, the sources of information about each method (health worker, friend, media, other), and whether the participant knew if his female intimate partner had ever used each method or was currently using it.

To assess knowledge of the contraceptive implant, participants were provided with a set of six statements regarding the safety (e.g., ‘Most women can safely use the implant’) and efficacy of the implant (e.g., ‘The implant is very effective in preventing pregnancy). A three-point Likert scale was used (agree, disagree, and unsure). The internal consistency of the knowledge composite scale was adequate (Cronbach’s Alpha: 0.64; 95% Confidence Interval (95% CI): 0.60–0.69).

To measure outcome expectancies of the contraceptive implant, participants were asked to imagine how it would be like for them if their female intimate partners were to use the implant. They were provided with eleven (11) statements each reflecting a positive or negative outcome expectancy (e.g., ‘I think that if my partner were to use the implant it would be very good because it lasts for a long time’ [positive] and ‘I think that if my partner were to use the implant, the implant would move around her body [negative]). A three-point Likert scale was used (agree, disagree, or unsure). Finally, to measure men’s support for their female intimate partners to use the implant in the future, participants were asked whether they would like their partners to use the implant in the future (response options: yes, no, or unsure). The implant was defined to the participants as a small plastic rod inserted under the skin of a woman’s arm to prevent pregnancy. A copy of the questionnaire with the full statements is provided as an additional file [Additional file 1: Questionnaire]. Participants were offered a nominal reimbursement, a chocolate bar, for their time taken completing the survey.

### Data analysis

Prior to analysis, items were rescored so that the valid knowledge statements and positive attitudes scored highest. Separate total scale scores were composed from the six items of knowledge composite and from the 11 items of the men’s outcome expectancy composite for the implant use by their female intimate partners. However, the scale scores were not used in the analyses due to the low internal reliability as indicated by the Cronbach’s Alpha statistic.

Data were analyzed using SPSS version 25. Descriptive statistics were performed to get an overview of the sample characteristics. A chi-square test was conducted to explore the associations between the demographic characteristics, knowledge scale, outcome expectancy items, and whether or not the men would like their female intimate partners to use the implant in the future. Significance level was set at p- value of equal to, or less than 0.05 (*p* ≤ 0.05). Prior to conducting the chi-square test, some variables were collapsed to become dichotomous variables. This was done in order to ascertain non-violation of the minimum expected cell frequency for the chi-square test. Thus, a 2 by 2 table design was used in this study.

## Results

A total of 65 men aged from 19 to 52 years with the mean (SD) of 31.2 (8.4) participated in this study. A small proportion, 23.1% (*n* = 15) were 24 years old or younger. Most had not completed secondary education (41.5%; *n* = 30) and 38.5% (*n* = 25) had completed secondary schooling. Only 15.4% (*n* = 10) had post-secondary school education. Most participants, 59.4%, (*n* = 38) reported they were unemployed. Most men, 87.2% (*n* = 56) were unmarried, but 81.3% (*n* = 52) reported to have a main sex partner and 43.8% (*n* = 28) reported to have a casual sex partner, while 18.8% (*n* = 12) did not have a main or casual partner. The reported reasons for being at the clinic on the day were for STI treatment by 61.5% (*n* = 40), 35.4% (*n* = 23) for an HIV test, and 4.6% (*n* = 3) for family planning.

Most participants, 76.6% (*n* = 49) endorsed the statement that both men and women should be responsible for family planning and 73.8% (*n* = 48) believed that men and women should decide together whether a woman should use contraception or not. Support for general contraception use by their female intimate partners was prevalent at 80.0% (*n* = 54). Awareness of the implant among men was relatively low, with 52.3% (*n* = 34) reporting they had never heard of the implant. Among those who had heard about the implant, 51.6% (*n* = 15) had heard about it from a friend, 32.3% (*n* = 10) from the healthcare worker and 32.3% (*n* = 10) from had heard about it from the media. Only 12.3% (*n* = 8) reported that their partners had used the implant before while the majority, 47.7% (*n* = 31) said their partners had never used the implant to their knowledge, and 38.5% (*n* = 25) said they did not know if their partners had ever used it. After having been informed by the data collector about the implant, 33.9% (*n* = 20) reported that they would like their partners to use the implant in the future, while 35.6% (*n* = 21) were unsure and 30.5% (*n* = 18) said they would not like their partners to use it.

Table 1 below compares men who reported they supported their partner using the implant in the future with those who did not support it or were unsure. Factors significantly associated with men’s support included men’s reports that their partner wished to have another child in future, knowledge that the implant is safe for use by women who have not had children, knowledge that the implant can effectively prevent pregnancy for 3 years, and the positive expectancy of the implant’s long-lasting effectiveness.

**Table 1:** Associations between selected demographic characteristics, knowledge and outcome expectancy of the implant against whether the men would like their female intimate partners to use the implant in the future, Chi-Square results

Variables	Would you like your partner to use the implant in the future^a^		
	Yes n (%)	No or unsure n (%)	Chi-Square (***p***-value)
**Demographics**			
Age group			
< 25 years old	3 (21.4%)	11 (78.6%)	
> 25 years old	17 (37.8%)	28 (62.2%)	
			1.27 (.26)
Education			
Up to incomplete secondary school	11 (40.7%)	16 (59.3%)	
Secondary school and post-secondary	9 (28.1%)	23 (71.9%)	
			1.04 (.31)
**Family planning responsibility and support**			
Who is responsible for FP			
Woman or man	5 (35.7%)	9 (64.3%)	
Both man and woman	15 (33.3%)	30 (66.7%)	
			.03 (.87)
Who should decide a woman should use FP			
Woman or man	7 (46.7%)	8 (53.3%)	
Both man and woman	13 (29.5%)	31 (70.5%)	
			1.46 (.23)
How do you feel about your partner using FP			
Support partner	17 (34.7%)	32 (65.3%)	
I don’t like it	2 (50.0%)	2 (50.0%)	
			.88 (.35)
Does your main partner/wife want a/another child			
Yes	14 (51.9%)	13 (48.1%)	
No or unsure	6 (18.8%)	26 (81.2%)	
			**7.16 (.01)**
**Knowledge composite**			
Most women can safely use the implant			
Agree	13 (50.0%)	13 (50.0%)	
Disagree or unsure	7 (21.2%)	26 (78.8%)	
			**5.38 (.02)**
Teenagers can safely use the implant			
Agree	13 (44.8%)	16 (55.2%)	
Disagree or unsure	7 (23.3%)	23 (76.7%)	
			3.04 (.08)
Women with no babies can safely use the implant			
Agree	11 (40.7%)	16 (59.3%)	
Disagree or unsure	9 (28.1%)	23 (71.9%)	
			1.04 (.31)
HIV positive women can safely use the implant			
Agree	11 (47.8%)	12 (52.2%)	
Disagree or unsure	9 (25.0%)	27 (75.0%)	
			3.26 (.07)
Implant is very effective in preventing pregnancy			
Agree	12 (40.0%)	18 (60.0%)	
Disagree or unsure	8 (27.6%)	21 (72.4%)	
			1.01 (.31)
Implant can prevent pregnancy for:			
About 3 years	9 (56.2%)	7 (43.8%))	
Don’t know or other^b^	11 (25.6%)	32 (74.4%)	
			**4.89 (.03)**
Implant protects against STIs and HIV			
Agree	4 (28.6%)	10 (71.4%)	
Disagree or unsure	16 35.6%)	29 (64.4%)	
			.23 (.63)
**Outcome expectancy composite**			
Implant could be easily inserted			
Agree	10 (29.4%)	15 (70.6%)	
Disagree or unsure	10 (29.4%)	24 (70.6%)	
			7.2 (.39)
Implant could be easily removed			
Agree	8 (38.1%)	13 (61.9%)	
Disagree or unsure	12 (31.6%)	26 (68.4%)	
			.26 (.61)
Implant is convenient			
Agree	14 (42.4%)	19 (57.6%)	
Disagree or unsure	6 (23.1%)	20 (76.9%)	
			2.43 (.12)
Implant lasts for long			
Agree	14 (46.7%)	16 (53.3%)	
Disagree or unsure	6 (20.7%)	23 (79.3%)	
			**4.44 (.04)**
I’d worry about her gaining weight if she uses the implant			
Agree	10 (43.5%)	13 (56.5%)	
Disagree or unsure	10 (27.8%)	26 (72.2%)	
			1.54 (.21)
I’d worry about her monthly bleeding if she uses the implant			
Agree	5 (41.7%)	7 (58.3%)	
Disagree or unsure	15 (31.9%)	32 (68.1%)	
			.41 (.52)
Implant could stop her from falling pregnant even after she removes it			
Agree	8 (50.0%)	8 (50.0%)	
Disagree or unsure	12 (27.9%)	31 (72.1%)	
			2.27 (.13)
Implant could move around her body			
Agree	4 (50.0%)	4 (50.0%)	
Disagree or unsure	16 (31.4%)	35 (68.6%)	
			1.07 (.30)
Implant could be painful to her			
Agree	4 (44.4%)	5 (55.6%)	
Disagree or unsure	16 (32.0%)	34 (68.0%)	
			.53 (.47)
Implant could harm her future babies			
Agree	1 (16.7%)	5 (83.7%)	
Disagree or unsure	19 (35.8%)	34 (64.2%)	
			.88 (.35)

^a^Only those who reported to have a partner

^b^Other includes 6 months, about a year, about 5 years, more than 5 years, unsure

Bold is significant

## Discussion

This study investigated factors associated with men’s support for the future use of the implant by their female intimate partners. Among the men, there were relatively low levels of awareness about the implant and minimal support for its future use by their female intimate partners. However, support for general contraceptive use was high among the men in this study. Factors associated with men’s support for the future use of the implant by their female intimate partners included: a) men knowing that the intimate partner wishes to have another child in future, b) knowing that the implant is safe for use by women who have not had children yet, c) knowing the timeframe within which the implant can effectively prevent pregnancy, and d) the perception that the implant last long. These findings are not unique, previous studies conducted elsewhere among men on women’s contraceptive use also identified a strong association between men’s awareness of contraceptive methods and their support for female intimate partner use [21–28]. Furthermore, men’s awareness of and support for modern contraceptive use was strongly associated with women’s desire to use contraception in Nigeria [21], and in Uganda [26]. Potentially, men may play an essential role in some women’s decision to use or not use contraception. Therefore, to improve men’s support for the use of the contraceptive implant by their female intimate partners, they need to be engaged with as well if efforts to improve the uptake of the implant among women in the country are to be realized. Thus, efforts to improve men’s knowledge and awareness of the implant could potentially improve women’s utilization of the contraceptive implant.

It is not clear why men who believe their partners want to have children in the future would be more likely to support the implant use. It is possible that the men’s fertility intentions were not aligned to their partner’s intentions, and they regarded the implant as an effective contraception. It is equally plausible that they supported their partner’s use of the implant because of its reversable attribute, enabling their partner to become pregnant when the couple so desired.

It is well known that more women than men attend health facilities for reproductive healthcare services including family planning, but very little is being done to improve men’s participation and involvement in reproductive health programmes in the country. Men do not come with their female intimate partners for family planning [5]. Therefore, most men, as well as some women generally have insufficient knowledge and understanding of female contraceptive methods, including the implant but men are hardly ever reached compared to women and continue to be distant in family planning programming. The findings of our study suggest the importance of actively engaging men in reproductive health and family planning programs, and the potential that men’s support could have on improving contraceptive use among women.

There are, of course, some important limitations in interpreting the findings of this study. The cross-sectional nature of the study being the first important one, cannot infer causality, meaning that improving men’s knowledge and awareness of the contraceptive implant will not necessarily result to improved uptake of the implant by the women. The other limitations are the small sample size of the men in this study, as well as the setting in which this study was undertaken and therefore, the findings cannot be generalizable. It is possible that most men were attending the “specialized” men only clinics where they will not be seen by female providers as well as not be amongst the many female patients who are filling up the waiting rooms in public health facilities [19]. Despite these limitations, the findings of this study highlight the need and importance of actively engaging men in reproductive health programming to improve contraceptive uptake and reduce the unmet need for contraception among women of reproductive age.

## Conclusion

Men’s perceptions about the implant’s safety and long-lasting characteristics are associated with their support for the future use of the implant by their female intimate partners. Thus, improving knowledge and awareness of the contraceptive implant among men may be a step in the right direction towards improving the uptake of the implant by women in the country. Unless men are well-informed and involved in reproductive health or family planning programming, support for their female intimate partners to use contraception will remain a challenge for many women. Interventions and strategies to engage men in family planning programs and enhance their participation in these programs are needed.

## Supplementary Information

**Additional file 1.**

## Data Availability

The dataset used for the current study is available from the corresponding author on reasonable request.

## Abbreviations

Cu IUD: Copper Intrauterine Device
HIV: Human Immunodeficiency Virus
IUD: Intrauterine device
LARC: Long-Acting and Reversible Contraceptives
STI: Sexually Transmitted Infections
SPSS: Statistical Package for the Social Sciences
TB: Tuberculosis
