# Supplementary material for: Factors affecting men’s support for the use of the contraceptive implant by their female intimate partners
Source: Contracept Reprod Med. 2020 Nov 23;5:36. doi: 10.1186/s40834-020-00140-7 (PMC7685537; doi:10.1186/s40834-020-00140-7)
Supplement: Supplementary file 1 — Additional file 1. [file 40834_2020_140_MOESM1_ESM.pdf]

______________ (1)

Entry 1: _____________

Entry 2: _____________

Cleaned ______________

Date: (2)	Staff (3)

IMBUMBA Survey 3-M

Ten Minutes of Your Time Can Help Improve Health Services

Thanks for taking time to fill out this survey.

This survey is completely anonymous.
Please do not put your name anywhere on it.

This survey is being conducted by
Professor Cathy Mathews
South African Medical Research Council
And
Professor Seth Kalichman
of the University of Connecticut in the United States

The information in this
survey will only be seen by the researchers.

Please answer all questions honestly.
Try not to skip any questions. You may stop doing the survey at anytime without penalty.

Please do not put your name on this survey

Thank you for agreeing to participate in this study. To remind you, this is a research study about contraception/family planning. These words are used to talk about ways to prevent pregnancy and to control when to have children. We want to learn more about your (and your partner's) contraceptive use and knowledge so that we can better help couples to make decisions about family planning.

PLEASE TELL US ABOUT YOURSELF BY MARKING OR TICKING YOUR ANSWER OR WRITING YOUR RESPONSE IN THE SPACE PROVIDED …

A1. What is your age?

_______________ years old

A2. Are you currently employed?

Yes	No

A3. What is your highest level of education?

	No formal education
	Attended primary school but did not complete
	Completed primary school
	Attended high school but did not complete
	Completed high school
	Attended university/technikon/college but did not graduate
	Graduated from university/technikon/college

A4. Do you have a main sexual partner (fasti)?

Yes	No

A5. Do you have any casual sexual partners (khwapheni)?

Yes	No

A6. Are you currently married?

Yes	No

A7. Are you living with your sexual partner or wife?

I do not have a sexual partner or wife	Yes	No

A10. Is your sexual partner or wife currently pregnant?

I do not have a sexual partner or wife	Yes	No	Unsure

A11. How many children do you have?

	None
	1
	2 or more

A12. When was your youngest child born?

	I do not have a child
	2016
	2015
	2014
	2013 or before

A14. Would you like to have a child/another child in the future?

Yes	No	Unsure

A15. When in the future do you think you would you like to have a child/another child?

	I do not want a child/another child
	During the next 12 Months
	12 - 24 months
	25 – 36 months
	More than 36 months
	Unsure

A16. Does your main partner or wife want a child /another child?

	I do not have a main partner or wife
	Yes
	No
	Unsure

A17. Why are you at the clinic today? Tick all that apply.

	To bring my baby for a checkup
	To get family planning
	For TB treatment
	For HIV treatment (antiretrovirals/ARVs)
	To get an HIV test
	To get treatment for an STI
	Other reasons (please specify): ______________________________

A18. Are you HIV positive?

	Yes
	No
	Don’t know
	Refuse to answer

NOW, PLEASE TELL US ABOUT THE FAMILY PLANNING INFORMATION AND SERVICES YOU'VE RECEIVED AT CLINICS OR DAY HOSPITALS…

B1. Have you ever visited a clinic for pregnancy prevention?

Yes	No

B2. In the **past year**, has a health worker/nurse talked to you about whether you want to have a child in the future?

Yes	No	Unsure

B3. In the **past year**, has a health worker/nurse told you about family planning methods that you and your partner can use to avoid having more children?

Yes	No	Unsure

B4. In the **past year**, did a health worker/nurse recommend that you and your partner use condoms along with another family planning method?

Yes	No	Unsure

THESE QUESTIONS ARE ABOUT USING FAMILY PLANNING. WE'RE INTERESTED IN UNDERSTANDING A BIT MORE ABOUT WHAT YOU AND YOUR PARTNER(S) THINK ABOUT FAMILY PLANNING AND HOW YOU AND YOUR PARTNER(S) USE IT. WE ARE INTERESTED IN YOUR OPINION. THERE ARE NO RIGHT OR WRONG ANSWERS

C1. Who do you think should be responsible for family planning?

The woman	Her male partner	Both the woman and her male partner

C2. Who do you think should decide whether a woman uses family planning?

The woman	Her male partner	Both the woman and her male partner

C3. When did you last have sex?

	I have never had sex
	In the past week
	In the past month
	In the past six months
	More than six months ago

C6. Have you and a partner ever used any method of family planning?

	I have never had sex
	Yes
	No
	Unsure

C7. How do you feel about your partner(s) using family planning?

	I do not have a partner
	I support my partner(s) using family planning
	I do not like my partner(s) to use family planning

C8. Have you ever discussed family planning with any of your main partners (fasti)?

I have never had a main partner	Yes	No

C9. Have you ever discussed family planning with any of your casual partners (khwapheni)?

I have never had a casual partner	Yes	No

C11. Have you ever given your main partner (fasti) **female condoms** to use when you are having sex to prevent pregnancy?

I have never had a main partner	Yes	No

BELOW ARE A FEW QUESTIONS ABOUT DIFFERENT TYPES OF FAMILY PLANNING METHODS. PLEASE ANSWER AS FULLY AS POSSIBLE. THINK ABOUT YOUR MOST RECENT PARTNER WHEN ANSWERING THESE QUESTIONS.

Implant:

F1. Have you heard about the **implant** (a small plastic rod inserted under the skin of a woman's arm to prevent pregnancy)?

Yes	No

F2. Where have you heard most of what you know about the implant?

	I have not heard about it
	Health worker
	Friends
	Media (radio, newspapers, the internet)
	Other (please specify): ___________________________

F8. To your knowledge, has your partner ever had an **implant**?

I have never had a partner	Yes	No	I don't know

F9. To your knowledge, does your partner currently have an **implant**?

I do not have a partner	Yes	No	I don't know

F10. Would you like your partner to use the **implant** in the future?

Yes	No	Unsure

Pill (oral contraceptive pill):

D1. Have you heard about family planning **pills**?

Yes	No

D6. To your knowledge, has your partner ever used family planning **pills**?

I have never had a partner	Yes	No	I don't know

D7. Would you like your partner to use family planning **pills** in the future?

Yes	No	Unsure

IUD:

E1. Have you heard about the intra-uterine device or **IUD** (an IUD is a loop or a coil inserted into a woman's uterus, by a health worker, to prevent pregnancy)?

Yes	No

E7. To your knowledge, has your partner ever had an **IUD**?

I have never had a partner	Yes	No	I don't know

E8. To your knowledge, does your partner currently have an **IUD**?	I do not have a partner	Yes	No	I don't know

E9. Would you like your partner to use the **IUD** in the future?	Yes	No	Unsure

F10. Would you like your partner to use the **implant** in the future?	I do not have a partner	Yes	No	I don't know

Injection:

G1. Have you heard about the **injection** (an injection that a health worker can give a woman to stop her from becoming pregnant)?	Yes	No

G7. To your knowledge, has your partner ever used the **injection**?	I have never had a partner	Yes	No	I don't know

G8. To your knowledge, does your partner currently use the **injection**?	I do not have a partner	Yes	No	I don't know

G9. Would you like your partner to use the **injection** in the future?	Yes	No	Unsure

Emergency contraception:

H1. Have you heard about **emergency contraception** (a pill that can be taken within 3 days after sex to prevent pregnancy)?	Yes	No

H3. Has your partner ever used **emergency contraception** after unprotected sex with you?

I have never had a partner	Yes	No	I don't know

Male condom:

I3. Have you ever used a male **condom** during sex?

I have never had sex	Yes	No

I4. Did you use a male **condom** the last time you had sex?

I have never had sex	Yes	No

Female condom:

J3. Has your partner ever used a **female condom** during sex with you?

I have never had sex	Yes	No	I don't know

J4. Did your partner use a **female condom** the last time you had sex?

I have never had sex	Yes	No	I don't know

NOW, WE'D LIKE TO KNOW WHAT YOU KNOW AND THINK ABOUT THE IMPLANT. (THE IMPLANT IS A SMALL PLASTIC ROD INSERTED UNDER THE SKIN OF A WOMAN'S ARM TO PREVENT PREGNANCY). THERE ARE NO RIGHT OR WRONG ANSWERS.

K1. Most women can safely use the implant

Agree	Disagree	Unsure

K2. Teenagers can safely use the implant

Agree	Disagree	Unsure

K3. Women who have not yet had a baby can safely use the implant

Agree	Disagree	Unsure

K4. Women with HIV can safely use the implant

Agree	Disagree	Unsure

K5. The implant is very effective at preventing pregnancy

Agree	Disagree	Unsure

K6. The implant can prevent pregnancy for

	A few months
	About 1 year
	About 3 years
	About 5 years
	More than 5 years
	Unsure

K7. The implant protects against STIs and HIV	Agree	Disagree	Unsure

FINALLY, WE'D LIKE TO KNOW WHAT IT WOULD BE LIKE FOR YOU IF YOUR PARTNER USED THE IMPLANT. IF YOU DO NOT HAVE A PARTNER, OR IF YOUR PARTNER HAS NEVER USED THE IMPLANT, PLEASE IMAGINE WHAT IT WOULD BE LIKE WHEN ANSWERING THE FOLLOWING QUESTIONS…

L12. I think an implant could be easily inserted	Agree	Disagree	Unsure
L13. The implant could be easily removed when my partner wants it to be	Agree	Disagree	Unsure
L14. The implant is convenient (my partner will not have to go to the clinic as often so it will save time)	Agree	Disagree	Unsure
L15. The implant is good because it lasts for a long time	Agree	Disagree	Unsure
L16. If my partner used the implant, I would worry about her gaining weight	Agree	Disagree	Unsure
L17. If my partner used the implant, I would be worried that her monthly bleeding would change or become irregular	Agree	Disagree	Unsure

L18. If she wanted to, my partner could get pregnant very soon after having the implant removed	Agree	Disagree	Unsure
L19. The implant could stop my partner from getting pregnant or make it harder to get pregnant in the future, even after it's taken out	Agree	Disagree	Unsure
L20. The implant could move around in my partner's body	Agree	Disagree	Unsure
L21. If my partner used the implant, it would be painful for her	Agree	Disagree	Unsure
L22. The implant could harm the babies my partner has in the future	Agree	Disagree	Unsure
L23. Overall, would you say your opinion about the implant is good or bad?	Good	Bad	I have no opinion about the implant

L24. Would you like to write anything else about the implant? For example, if you think there are good or bad things about this method of family planning, or any questions you might like to ask?

____

____
